# Functional connectivity of cognition-related brain networks in adults with fetal alcohol syndrome

**DOI:** 10.1186/s12916-023-03208-8

**Published:** 2023-12-13

**Authors:** Benedikt Sundermann, Reinhold Feldmann, Christian Mathys, Johanna M. H. Rau, Stefan Garde, Anna Braje, Josef Weglage, Bettina Pfleiderer

**Affiliations:** 1 Institute of Radiology and Neuroradiology, Evangelisches Krankenhaus Oldenburg, Universitätsmedizin Oldenburg, Oldenburg, Germany; 2 Clinic of Radiology, Medical Faculty, University of Münster, Albert- Schweitzer-Campus 1, Building A1, 48149 Münster, Germany; 3 Research Center Neurosensory Science, Carl von Ossietzky Universität Oldenburg, Oldenburg, Germany; 4 Department of General Pediatrics, University Hospital Münster, Münster, Germany; 5 Department of Neurology With Institute of Translational Neurology, University Hospital Münster, Münster, Germany

**Keywords:** Fetal alcohol syndrome, Prenatal alcohol, Executive functions, Higher criticism, Connectivity, Resting-state fMRI

## Abstract

**Background:**

Fetal alcohol syndrome (FAS) can result in cognitive dysfunction. Cognitive functions affected are subserved by few functional brain networks. Functional connectivity (FC) in these networks can be assessed with resting-state functional MRI (rs-fMRI). Alterations of FC have been reported in children and adolescents prenatally exposed to alcohol. Previous reports varied substantially regarding the exact nature of findings. The purpose of this study was to assess FC of cognition-related networks in young adults with FAS.

**Methods:**

Cross-sectional rs-fMRI study in participants with FAS (*n* = 39, age: 20.9 ± 3.4 years) and healthy participants without prenatal alcohol exposure (*n* = 44, age: 22.2 ± 3.4 years). FC was calculated as correlation between cortical regions in ten cognition-related sub-networks. Subsequent modelling of overall FC was based on linear models comparing FC between FAS and controls. Results were subjected to a hierarchical statistical testing approach, first determining whether there is any alteration of FC in FAS in the full cognitive connectome, subsequently resolving these findings to the level of either FC within each network or between networks based on the Higher Criticism (HC) approach for detecting rare and weak effects in high-dimensional data. Finally, group differences in single connections were assessed using conventional multiple-comparison correction. In an additional exploratory analysis, dynamic FC states were assessed.

**Results:**

Comparing FAS participants with controls, we observed altered FC of cognition-related brain regions globally, within 7 out of 10 networks, and between networks employing the HC statistic. This was most obvious in attention-related network components. Findings also spanned across subcomponents of the fronto-parietal control and default mode networks. None of the single FC alterations within these networks yielded statistical significance in the conventional high-resolution analysis. The exploratory time-resolved FC analysis did not show significant group differences of dynamic FC states.

**Conclusions:**

FC in cognition-related networks was altered in adults with FAS. Effects were widely distributed across networks, potentially reflecting the diversity of cognitive deficits in FAS. However, no altered single connections could be determined in the most detailed analysis level. Findings were pronounced in networks in line with attentional deficits previously reported.

**Supplementary Information:**

The online version contains supplementary material available at 10.1186/s12916-023-03208-8.

## Background

Prenatal alcohol exposure (PAE) can negatively affect a wide range of cognitive functions throughout life. These functions include general intelligence, attention, executive functions (including inhibitory control), learning and memory, language, mathematical abilities, social cognition [1, 2], and impulse control [1, 3, 4]. Deficits associated with PAE frequently persist into adulthood [5, 6]. While the term fetal alcohol spectrum disorders (FASD) generally encompasses a broad range of possible conditions related to PAE, only the full picture of characteristic physical (including growth retardation and facial abnormalities), psychological, and cognitive features is termed fetal alcohol syndrome (FAS) [2, 7, 8].

Brain activity in individual regions underlying specific cognitive functions can be assessed by functional neuroimaging with a wide range of targeted tests [9]. However, a frequent neuroscientific observation is that many higher cognitive functions, such as those negatively affected in FAS, are subserved by activity in few common sets of brain regions, i.e., functional brain networks. These include task-positive networks with overlapping definitions such as the central executive network, cognitive control network, or multiple demands network as well as networks closely interacting with them, such as the salience network and default mode network [10–12]. Spontaneous activity and functional connectivity within and between these networks can be examined by resting-state functional magnetic resonance imaging (rs-fMRI) [13, 14]. Static functional connectivity (FC) analysis methods identify correlated activity over a full rs-fMRI data acquisition period [14]. They have recently been supplemented by approaches for assessing dynamic or time-varying FC. Such dynamic FC analyses promise a deeper understanding of dynamic interactions of brain regions within and across these functional networks [15–17] in health and disease. Dynamic FC measures have been associated with differences in individual attentional performance [18, 19] and impulsivity [20, 21]. For example, altered FC dynamics have been observed in participants with attention deficit hyperactivity disorder (ADHD) [22–24].

Few studies have directly investigated resting-state FC within and between brain networks related to higher cognitive functions in individuals with FAS or prenatal alcohol exposure: Focusing on within-network connectivity, Fan et al. observed reduced FC in a subset of regions within the default mode, salience, ventral attention, dorsal attention, and right fronto-parietal executive control networks in children with FASD compared with non-exposed controls. These networks reflect cognitive functions typically affected in children with FASD. Networks not directly related to cognition were, however, not affected [25]. In another study with children and adolescents with FASD, Little et al. mainly described reductions of FC between core regions of the salience and fronto-parietal control networks and regions from other cognition-related networks rather than within networks [26]. In contrast, Ware et al. found lower within-network but higher between-network FC in attention-related networks in children with FASD [27]. All three studies report relatively high overall similarity of FC in cognition-related networks between exposed individuals and controls, while FC group differences had relatively small effect sizes, contrasting with the distinct clinical deficits in these individuals [25–27]. Further rs-fMRI and methodologically related FC studies in individuals with PAE report evidence of altered overall functional brain organization based on global graph-theoretical measures [28–31] as well as FC alterations of the default mode network [32] and within networks less directly related to cognitive control [33, 34]. No previous study has, however, explored dynamic FC in FASD. Beyond that, amid studies describing persisting cognitive deficits into adulthood [5, 6], FC has not been previously examined in adults prenatally exposed to alcohol.

The main goal of this study was therefore to examine functional connectivity in cognition-related functional brain networks in young adults with FAS and to assess whether these patterns are comparable to alterations previously observed in affected children and adolescents.

The following hypotheses should be tested:

- Static FC in the connectome of all brain regions constituting cognition-related brain networks is altered in young adults with FAS compared with a control group without prenatal alcohol exposure (omnibus test, bi-directional effects possible).
- Static FC within individual cognition-related brain networks is altered in FAS participants compared with controls without prenatal alcohol exposure (bi-directional effects possible).
- Static FC between cognition-related brain networks is altered in FAS participants compared with controls without prenatal alcohol exposure (bi-directional effects possible).

In an additional exploratory analysis, we addressed dynamic interactions between cognition-related brain regions in FAS participants compared with non-exposed controls. This dynamic FC analysis [17] focused on transitions between putative FC states. Rationale of this analysis is that less stable FC states in FAS might underlie impaired impulse control (similar to reports in ADHD [22–24]).

## Methods

The study was carried out in a research setting outside routine clinical care. Samples overlapped with previously published task-based fMRI studies on inhibitory control with joint data acquisition in a superordinate research project: a control group overlap with a task-based study in phenylketonuria [35] and a female patient and control group overlap with a task-based study in FAS [36]. There was no sample overlap with previous rs-fMRI research in FAS or FASD.

### Participants

Young adult persons (*n* = 50) with FAS seen for regular appointments at the FAS outpatient clinic at the Children’s healthcare center Haus Walstedde (Drensteinfurt, Germany) were initially invited based on standardized primary inclusion and exclusion criteria: These primary inclusion criteria were a diagnosis of FAS made by a specialist based on the Majewski criteria [37] (see supplementary Additional_file_1.pdf: supplementary methods) [37, 38], and being 18 to 32 years of age. Primary exclusion criteria were contraindications for MRI, severe psychiatric (e.g., current symptomatic episode of major depression, bipolar disorder or schizophrenia), neurological (e.g., stroke or epilepsy) or medical (e.g., cancer) conditions, pregnancy, and severe sensory impairments. Less severe psychiatric comorbidity or symptoms in general (e.g., signs of hyperactivity or a history of adjustment disorder) as well as medication in general were not defined as primary exclusion criteria for the FAS group since they are common in individuals with a history of prenatal alcohol exposure [39]. In four participants, no fMRI data could be acquired because of claustrophobia. After data acquisition, further participants were excluded after review of potentially biasing medication, structural brain lesions, and MRI data quality control before any FC group analyses: Two participants were excluded from the analyses due to use of potentially psychoactive anti-allergic medication unrelated to FAS, which can alter rs-fMRI measurements [40]. One subject was excluded because of a callosal hypoplasia leading to structural image misregistration. Data from further 4 FAS participants were excluded because of excessive head motion (based on motion parameters, see section “Pre-processing and image quality control” for criteria). All results are based on the remaining 39 FAS participants. Current intake of the following potentially psychoactive medication was reported in the FAS group: methylphenidate or derivatives (*n* = 8), antipsychotics (*n* = 5), and antidepressants (*n* = 1).

The control group consisted of participants without a history of prenatal alcohol exposure. Initially, *n* = 52 participants were recruited by using the internal information board for employees of the Münster university hospital and mailing lists of medical students at the Münster medical faculty. Apart from general exclusion of participants with psychoactive medication in the control group, inclusion and exclusion criteria (both for initial inclusion and after data acquisition) were identical in both groups. Reasons for exclusion after data acquisition in this group were: Two participants were excluded due to use of psychoactive medication (disclosed at the study appointment). One subject was excluded because of a large frontal venous anomaly potentially biasing fMRI data [41]. One fMRI dataset was excluded because of a technical failure. Data from 4 participants were excluded because of excessive head motion (3 based on motion parameters, 1 based on visual quality indicators, see section “Pre-processing and image quality control” for criteria). We observed a statistically significant age difference between the groups with a small effect size (Table 1). We refrained from excluding further control participants in order not to compromise statistical power considering that only small age effects on FC are expected in this particular age range [42]. Finally, 44 controls were included in further analyses. Demographical data of the final sample are reported in Table 1.

**Table 1:** Demographical and clinical characteristics of participants with FAS and controls

**a)**	Group	Total	Female	Male	*p*^a^
Sex	FAS	39	17 (43.6%)	22 (56.4%)	.319
	CON	44	24 (54.5%)	20 (45.5%)	
**b)**	Group	Mean ± SD	Median	Range	*p*^b^
Age (years)	FAS	20.9 ± 3.4	20	18–32	.013*
	CON	22.2 ± 3.4	21	18–32	
EHI Handedness index	FAS	71.6 ± 37.9	83.3	− 58.3–100	.887
	CON	69.8 ± 40.3	83.3	− 58.3–100	
TMT reaction time (sec)	FAS	97.2 ± 36.7	86.3	54.3–220.0	< .001*
	CON	54.0 ± 8.7	54.1	35.5–74.8	
IQ (mean ± SD)^c^	FAS^d^	82.0 ± 16.7	80.5	58–116	< .001*
	CON	117.7 ± 15.0	116.5	88–150	

^a^Chi-squared test (sex distribution between groups)

^b^Mann–Whitney *U* test

^c^based on TMT reaction times

^d^ only calculated for *n* = 36 within valid range

*significant difference

*FAS* fetal alcohol syndrome, *CON* controls, *SD* standard deviation, *NA* not applicable, *EHI* Edinburgh Handedness Inventory, *TMT* trail-making task, *IQ* intelligence quotient estimated from TMT performance

### Neuropsychological pre-assessment

Participants completed questionnaire-based pre-tests for handedness (Edinburgh Handedness Inventory, EHI) [43], processing speed (trail-making task, TMT) [44], and screening for severe mental comorbidity (DIA-X Stamm-Screening questionnaire, SSQ) [45]. A general intelligence estimate was calculated based on TMT results [44, 46]. Quantitative test results are presented in Table 1.

### Acquisition of MRI data

MRI data were acquired at 3 Tesla (Intera with Achieva upgrade, Philips, Best, NL) using a single-channel transmit/receive head coil. FMRI data were acquired during 9:45 min of wakeful rest using gradient echo planar imaging covering the whole brain (234 functional volumes after 5 non-recorded dummy scans to allow for signal equilibration; repetition time: 2500 ms, echo time: 35 ms, 36 axial slices, spatial resolution 3.6 × 3.6 × 3.6 mm). Participants were instructed to keep their eyes open and think of nothing in particular during the rs-fMRI acquisition. T1-weighted 3D data were acquired with an inversion-prepared turbo field echo (TFE) sequence (inversion time: 411 ms, repetition time: 7.1 ms, echo time: 3.5 ms, flip angle: 9°, sagittal slices measured with 2 mm thickness, reconstructed spatial resolution by zero-filling in k-space 1.0 × 1.0 × 1.0 mm).

### Analysis of MRI data

#### Pre-processing and image quality control

MRI data were converted to the Brain Imaging Data Structure (BIDS) [47] using in-house scripts preceding the BiDirect-BIDS-ConverteR [48, 49]. Facial features were removed from the T1-weighted anatomical data [50]. Main MRI data pre-processing was carried out using fMRIPrep [51] (version 20.0.7, RRID:SCR_016216) briefly consisting of motion estimation and correction, co-registration of fMRI and structural MRI data, estimation of noise regressors, and standard space normalization. Please consult the Supplementary Methods for further details. Subsequent actual denoising was carried out using fMRIDenoise [52] (version 0.2.1), comprising regressing out 24 head motion parameters (3 translations, 3 rotations, their 6 temporal derivatives, and their 12 quadratic terms) [53], 8 physiological noise parameters (mean physiological signals from white matter and cerebrospinal fluid, their 2 temporal derivatives, and 4 quadratic terms) [53] as well as movement spike regression based on frame-wise displacement (FD > 0.5 mm) and so-called “DVARS” (> 3) thresholds [54], temporally filtering (0.008–0.08 Hz), and, finally, smoothing the resulting standard space image with a Gaussian kernel (FWHM = 6 mm). This pre-processing leads to denoised fMRI data in a common standard space as input for further analyses.

The following steps were taken for MRI data quality control (numbers of excluded participants reported in the section “participants”): Structural MRI data were screened by a radiologist for incidental findings and major artifacts. fMRIPrep reports were reviewed for registration errors and image artifacts (including signs of strong motion artifacts in the carpet plots). Subject exclusion for excessive head motion (see section “Participants”) was based on pre-processing criteria (mean frame-wise displacement, FD > 0.3 mm or maximum FD > 5 mm or more than 20% outlier data points). FD did not differ significantly between FAS and controls. However, there was a trend towards higher mean and maximum FD in the FAS group (see Supplementary Table 1).

#### Static functional connectivity analysis: general approach

Functional connectivity analyses were based on a cortical atlas (“Schaefer atlas”) derived from rs-fMRI data in 1489 participants. The atlas was obtained from TemplateFlow (RRID:SCR_021876) to match the dimensions of the fmriprep outputs [55]. The atlas version with 400 parcels adopted here is the most extensively validated version of this atlas, e.g., regarding stability and correspondence with markers of brain function [56]. The individual parcels in the published atlas have been matched to 17 non-overlapping networks from a previously established atlas by Yeo et al. [57]. Ten cognition-related components out of these 17 networks were selected for further analysis: the dorsal attention network (2 sub-networks A and B), the salience / ventral attention network (2 sub-networks A and B), the mainly fronto-parietal control network (3 sub-networks A-C), and the default mode network (3 sub-networks A-C). Time-series extraction from the pre-processed fMRI data and calculation of *z*-transformed Pearson correlation coefficients as primary measures of FC were carried out with the Data Processing Assistant for Resting-State fMRI (DPARSF, version 5.2, RRID:SCR_002372) [58] based on Matlab 2019b (The MathWorks, Natick, MA, USA, RRID:SCR_001622). As a basis for subsequent modelling, we carried out multiple linear models (one model per pair of regions), comparing *z*-transformed correlation coefficients (dependent variable) among regions of interest between FAS participants and controls. The following independent variables were included in the multiple linear regression models: group (FAS vs. control, categorical), sex (categorical), age (normalized to center: 0, and standard deviation: 1), mean FD, and a constant term (intercept). Main effects of the factor group (FAS vs. controls, two-sided) are the basis of the subsequent analyses at different spatial scales. A previous analogous analysis based on multiple two-tailed two-sample *t*-tests, i.e., not including age, sex, and head movement with similar results has been made available in the preprint version of this article [59].

We followed a hierarchical statistical testing approach with three levels of analysis: (1) first, determining whether there is any alteration of FC in FAS participants compared with controls in the full connectome of 243 regions constituting these 10 cognition-related networks (i.e., an omnibus test) globally and subsequently aiming to resolve these findings, (2) to the level of either FC within each network or between-network connectivity, and finally (3) to individual connections.

#### Static functional connectivity analysis: global analysis

The omnibus test on the full connectome (main effect of group: FAS vs. control) is based on the “Higher Criticism” (HC) approach [60] in an improved version [61] implemented in Matlab (https://www.stat.cmu.edu/~jiashun/Research/software/HC/). HC statistics can be applied in order to test whether there are any non-zero effects within a large number of individual tests carried out in high-dimensional datasets. They are thus suitable to identify the existence of rare and weak effects in such data [60]. HC follows the rationale of *p*-value histogram analyses: Under a null-hypothesis of only zero effects in multiple parallel tests, an equal distribution of *p*-values is expected. Under the alternative hypothesis of existing non-zero effects, there is an excess of low *p*-values [62]. In simplified terms, HC statistics quantitatively test a joint hypothesis of such an excess of low *p*-values [60, 62]. HC has been increasingly popular for detecting effects in high-dimensional data such as in genetic [60] and economic [63] research. It has been argued that HC could be favorable for the detection of rare events compared with conventional false discovery rate (FDR) or family-wise error rate (FWE) correction methods [60]. Considering the similarly high dimensionality of FC datasets, HC has recently been applied to rs-fMRI analyses [64, 65]. Both, in this global analysis and the subsequent network-wise HC-based hypothesis tests, we used *p*-value histograms (main effect of group) as a plausibility check.

#### Static functional connectivity analysis: within-network HC analysis

Subsequently, we aimed to determine which of the 10 cognition-related networks were affected by within-network functional connectivity differences between FAS and control participants. We therefore carried out equivalent HC tests separately for these networks. Each set of tests included the full set of correlation coefficients between all regions within each network.

#### Static functional connectivity analysis: between-network HC analysis

For a similar analysis of between-network FC (i.e., to determine whether any between-network FC differences were present), we concatenated all parcels for each of the 10 networks separately. This resulted in a single mask for each network before time-series data extraction. The resulting correlation coefficients were assessed with an equivalent HC test.

#### Static functional connectivity analysis: analysis of individual connections

In a third level, we aimed to identify single between- and within-network connections exhibiting statistically significant FC differences between FAS participants and controls. Therefore, in contrast to the previously described HC-based joint hypothesis tests, we now FDR-adjusted [66] the individual hypothesis tests (main effect of group: FAS vs. control) of between- and within-network connectivity (*q* < 0.05), using an FDR implementation in Matlab (https://brainder.org/2011/09/05/fdr-corrected-fdr-adjusted-p-values). This was carried out separately for either all 45 between-network connections or all individual connections, and also within each separate network.

#### Exploratory time-resolved functional connectivity analysis

Beyond the static FC analysis, we carried out an exploratory dynamic FC analysis, using a sliding window approach with the DynamicBC toolbox (version 2.2) [67]. FC between all 243 regions in the cognition-related networks was calculated for each individual subject by Pearson linear correlation separately in overlapping windows with a length of 18 consecutive functional volumes equivalent to 45 s, similar to window lengths in previous studies [68, 69], and with a 60% overlap. The resulting time-resolved FC estimates from individual time windows were grouped by similarity (*K*-means cluster analysis, distance measure: correlation) in order to derive presumed FC states in the entire sample. Established methods were used to estimate the optimal number of clusters and assess the goodness of fit of the clustering solutions: (1) The optimal number of clusters (search range: 2 to 10) was estimated using the Calinski-Harabasz [70] and Davies–Bouldin [71] indices resulting in 2 clusters (see Supplementary Fig. 1). (2) Cluster-separability was estimated by a silhouette analysis. Briefly, the silhouette analysis assesses, how similar an individual element is to other elements within its own cluster compared with elements in other clusters [72]. The following summary measures describing the temporal dynamics of putative FC states were calculated for individual participants: number of transitions (NT) between connectivity states, mean dwell time (MDT) per cluster, and frequency of observing each cluster (FRC). Participants with FAS and controls were compared regarding NT and MDT using *t*-tests or Mann–Whitney *U* tests.

### Statistical analysis

Statistical modelling is inherent to the FC analyses described above and is thus presented within the preceding sub-sections. Statistical tests on clinical and demographical data were carried out in SPSS (version 27.0, IBM, Armonk, NY, USA, RRID:SCR_002865).

## Results

### Static functional connectivity analysis: global analysis

**Fig. 1:**
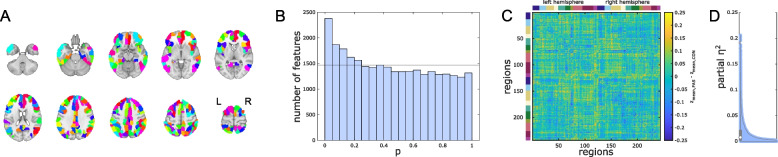
Global analysis of static functional connectivity of cognition-related brain networks. **A** Full atlas-based selection of 243 individual brain regions in cognition-related networks (redundant color coding for illustration of atlas resolution only). **B** *P*-value histogram of multiple individual linear models (main effect of group) comparing functional connectivity among all these brain regions between FAS patients and control participants. Under the null hypothesis of equal functional connectivity in both groups, equal numbers of *p*-values are expected in each histogram bin. The histogram shows an excess of low *p*-values. The existence of at least rare and/or weak effects visualized in the histogram is confirmed by a quantitative test of the joint hypothesis based on higher criticism statistics, following the same rationale. This means that regarding a significant number of functional connections, FAS patients differ from healthy controls. **C** Unthresholded matrix of connectivity group differences describing the full connectome of cognition-related brain regions. Yellow: mean *z*-transformed correlation coefficients relatively increased in FAS compared with controls. Blue: relatively decreased functional connectivity in FAS. Color coding of networks identical with Fig. 4. **D** Standardized effect size estimates (partial *η*^2^ from linear models)

We observed significantly altered FC of cognition-related brain regions in FAS participants compared with non-exposed controls in the global (all parcels of all 10 cognition-related networks) analysis over the entire data acquisition period. This finding is based on a joint hypothesis test (HC test statistic: 25.80, a higher value represents stronger support for an excess of low *p*-values in the underlying primary hypothesis tests). This joint hypothesis test aims to detect the existence of alterations within this high-dimensional dataset but which does not identify which exact connections are altered (Fig. 1). Quantitative results of FC group differences in connections between single atlas regions (Additional_file_2.csv, Additional_file_3.csv) as depicted in Fig. 1C are shared together with standardized effect size estimates (Additional_File_4.csv).

### Static functional connectivity analysis: within-network HC analysis

**Fig. 2:**
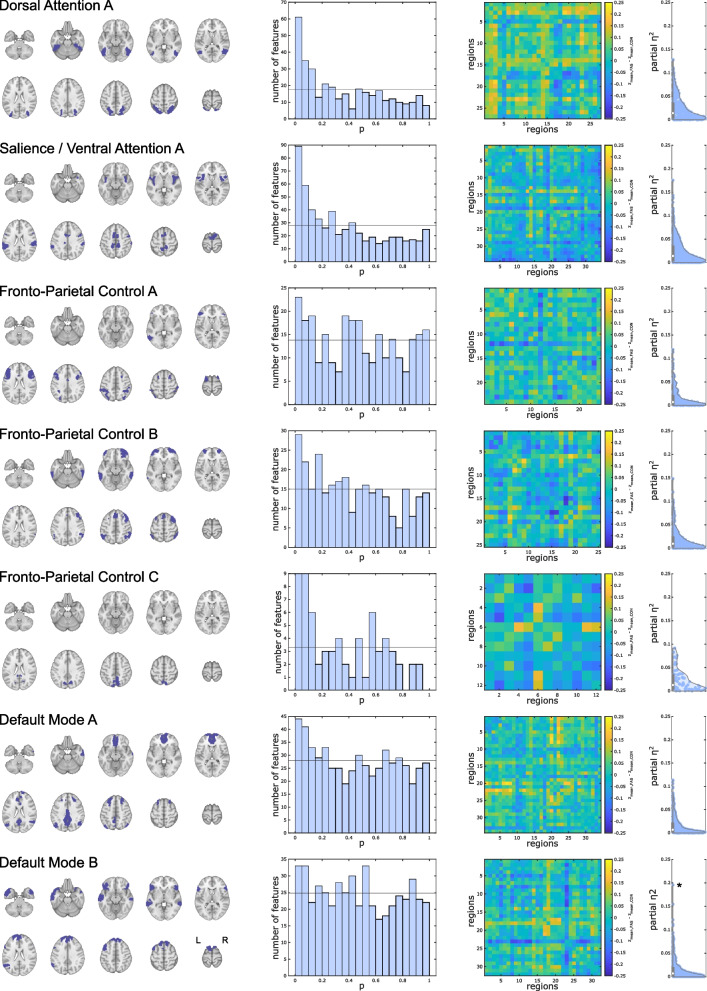
Within-network static functional connectivity differences in cognition-related brain networks. Seven (out of ten) sub-networks exhibiting altered functional connectivity in FAS patients compared with controls. First column: Overview of the networks’ overall extent. Second column: *P*-value histograms (different scaling reflecting different numbers of regions in each network) of multiple linear models (main effect of group) comparing functional connectivity within these sub-networks between FAS patients and control participants. Under the null hypothesis of equal functional connectivity in both groups, equal numbers of *p*-values are expected in each histogram bin. The histograms show an excess of low *p*-values, quantitatively confirmed by a test of the joint hypothesis based on higher criticism statistics. This means that FAS patients differ from healthy controls regarding at least rare and/or weak effects. Third column: Unthresholded matrices of connectivity group differences describing the full connections of cognition- related brain regions within each network. Yellow: mean *z*-transformed correlation coefficients relatively increased in FAS compared with controls. Blue: relatively decreased functional connectivity in FAS. Fourth column: Standardized effect size estimates (partial *η*^2^ from linear models, * single connection with significant group difference, false-discovery-rate-corrected *p* < 0.05 within the network but not significant when correcting across all connections). The remaining three networks without significant results are presented in Fig. 3

**Fig. 3:**
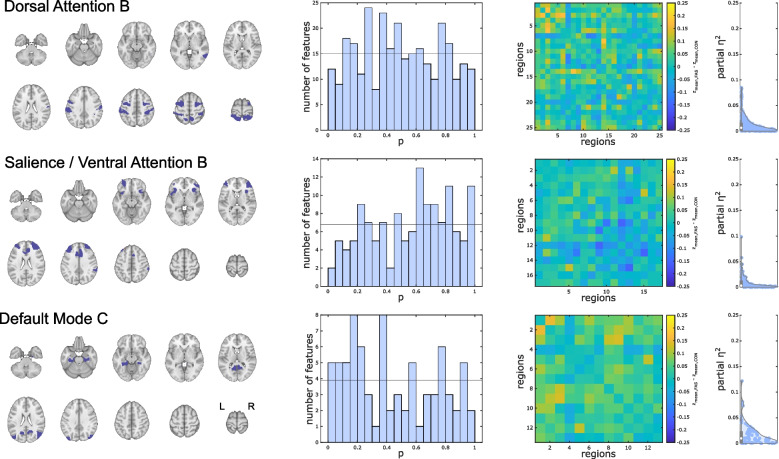
Within-network static functional connectivity of cognition-related brain networks (networks without significant group differences). Three (out of ten) sub-networks not exhibiting altered functional connectivity in FAS patients compared with controls. First column: Overview of the networks’ overall extent. Second column: *P*-value histograms of multiple linear models (main effect of group) comparing functional connectivity within these sub-networks between FAS and control participants (global null hypothesis not rejected based on HC test statistic). Third column: Unthresholded matrix of connectivity group differences describing the full connections of cognition-related brain regions within each network. Yellow: mean *z*-transformed correlation coefficients relatively increased in FAS compared with controls. Blue: relatively decreased functional connectivity in FAS. Fourth column: Standardized effect size estimates (partial *η*^2^ from linear models)

FC was altered within 7 out of 10 of these cognition-related brain networks based on the joint hypothesis tests. Based on the HC test statistic and supported by *p*-value histograms of tests of individual functional connections, this effect was most obvious in the salience / ventral attention A network (HC test statistic: 13.31), followed by the dorsal attention A sub-network (HC test statistic: 11.27). However, findings also spanned across sub-networks A, B, and C of the fronto-parietal control (HC test statistics: 4.02, 5.20, and 4.82) and sub-networks A, and B of the default mode (HC test statistics: 4.56 and 3.45) networks (Fig. 2). Three networks did not exhibit significant group effects: the dorsal attention B (HC test statistic: 1.00), salience/ventral attention B (HC test statistic: − 1.15), and default mode C (HC test statistic: 2.84) components (Fig. 3).

### Static functional connectivity analysis: between-network HC analysis

**Fig. 4:**
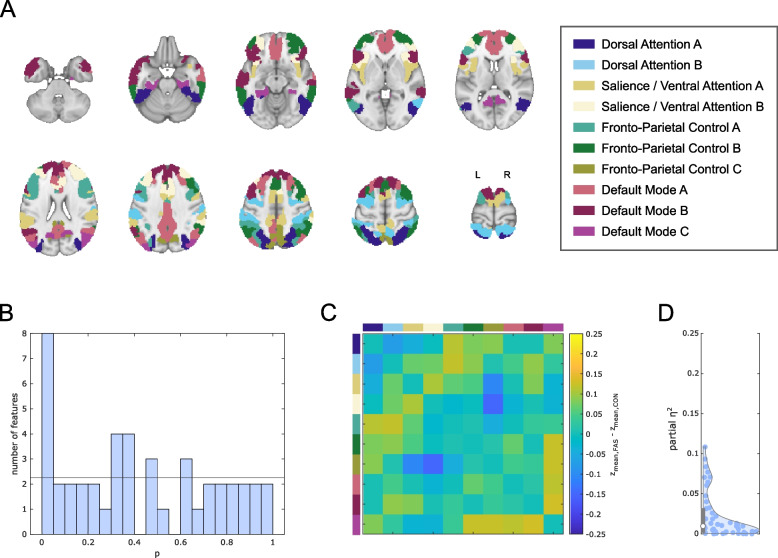
Between-network static functional connectivity of cognition-related brain networks. **A** 10 cognition-related networks. Each color represents an individual sub-network (network-wise concatenation of individual regions based on atlas labels). **B** *P*-value histogram of multiple linear models (main effect of group) comparing functional connectivity among these sub-networks between FAS patients and control participants. Under the null hypothesis of equal functional connectivity in both groups, equal numbers of *p*-values are expected in each histogram bin. The histogram shows an excess of low *p*-values quantitatively confirmed by a test of the joint hypothesis based on higher criticism statistics. This means that regarding a significant number of functional connections, FAS patients differ from healthy controls. **C** Unthresholded matrix of connectivity group differences describing the full connectome of cognition- related brain regions. Yellow: mean *z*-transformed correlation coefficients relatively increased in FAS compared with controls. Blue: relatively decreased functional connectivity in FAS. **D** Standardized effect size estimates (partial *η*^2^ from linear models)

A subsequent analysis revealed altered FC between cognition-related brain networks based on an equivalent joint hypothesis test (HC test statistic: 5.95). Descriptively, underlying strongest relative decreases of FC (ranking of correlation coefficient group differences) in FAS participants were observed between the salience / ventral attention B and fronto-parietal control C sub-networks. The strongest relative increases were observed between the dorsal attention B and fronto-parietal control sub-networks as well as between the default mode C sub-network and other parts of the default mode network and fronto-parietal control network (Fig. 4).

### Static functional connectivity analysis: analysis of individual connections

A single connection within the default mode B sub-network between the left dorsal prefrontal cortex and the right temporal lobe (decreased FC in FAS, difference of group-averages *z*-transformed correlation coefficients: − 0.210, partial *η*2 = 0.196) was statistically significant with FDR-adjustment within this network only (*p* = 0.0198), however not when adjusting (FDR) across all connections. None of the other individual FC alterations (either from the within- or between-network analysis) were statistically significant when correcting the separate tests of individual connections for multiple comparisons.

### Exploratory time-resolved functional connectivity analysis

In the exploratory dynamic FC analysis, a solution consisting of two FC states was empirically derived as the optimal number of clusters across the entire sample. These two clusters representing putative FC states were only weakly separable (see silhouette values and further goodness-of-fit statistics for the clustering solutions in Supplementary Fig. 1). Both clusters differed mainly regarding (1) the relatively connectivity strength of the DMN and (2) the extent that the DMN appeared interconnected with other cognition-related brain networks. See Additional_file_1.pdf: Supplementary Fig. 2 for further details on the clusters. FAS participants did not differ significantly regarding the temporal dynamics of these FC states NT, MDT, and FRC (Table 2).

**Table 2:** Group comparison results for the optimal clustering solution (2 cl) of the time-resolved functional connectivity analysis

	FAS	CON	*p*
Number of transitions^a^	6.59 ± 2.99	7.11 ± 2.24	0.366
Mean dwell time (cluster 1)^b^	3.33 (0.00–13.5)	3.29 (1.33–27.00)	0.809
Mean dwell time (cluster 2)^b^	3.00 (1.00–28.00)	3.00 (1.00–9.00)	0.982
Frequency (cluster 1)^c^	57.14 (0–96) %	53.57 (11–96) %	0.780
Frequency (cluster 2)^c^	42.86 (4–100) %	46.43 (4–89) %	0.780

Clusters represent estimates of putative FC states. In summary, cluster 1 represents widely distributed FC dominated by the DMN while cluster 2 exhibits stronger dichotomization between the DMN and the other cognition-related networks

^a^mean ± standard deviation and *t*-test result)

^b^ unit: number of (partially overlapping) windows, median (range) and Mann–Whitney *U* test result

^c^ median (range) and Mann–Whitney *U* test result

*FAS* fetal alcohol syndrome, *CON* controls

## Discussion

In summary, FC was altered in adults with FAS compared to controls not exposed to alcohol prenatally both within a majority of cognition-related networks (including the salience / ventral attention networks and dorsal attention networks, as well as, to a lesser degree, the fronto-parietal control network, and the default mode network) and between these networks. These results on the global and network level are based on an HC approach, indicating that at least rare and weak group effects seem to be present [60]. HC-based findings do, however, not necessarily mean that FC is changed in a majority of connections. Group effects could not be further resolved to connections between individual regions using conventional mass-univariate testing with multiple-comparison correction. In the additional exploratory time-resolved FC analysis, altered FC dynamics in the FAS group could not be observed.

### Wide distribution of group effects across networks

The wide distribution of findings across cognition-related networks is in line with the similarly wide range of cognitive deficits observed in individuals with FAS [1]. It thus suggests a rather distributed neural basis (i.e., unspecific alcohol-related damage) of such deficits rather than strongly localized alterations. A similarly wide distribution of FC alterations across networks was observed by Fan et al. in children with FASD [25]. Thus, this observation of a wide distribution spans different age groups (from childhood to young adulthood) and rs-fMRI analysis approaches. Visual interpretation of the connectivity matrices reveals different directionalities of findings: Some connections exhibited higher, and some lower FC in FAS. This bidirectionality is generally in line with a study in children with FASD by Ware et al.. They also report different directionalities of FC alterations [27]. Consequently, a simple picture of either overall increases or decreases of FC in FAS does not exist.

### Group differences in attention-related networks

The observation of more obvious effects in attention- and salience-related systems compared with networks underlying other cognitive functions highlights the importance of attentional deficits in FASD [1, 73–77], including adults [5, 78]. However, this interpretation might be limited by the infeasibility of a direct quantitative comparison between networks as well as reverse inference [79, 80]. Our rs-fMRI findings are generally in line with previous studies suggesting a particular involvement of attentional functions and underlying neural systems in FAS when compared to other cognitive deficits: Response and activation patterns in a Go/NoGo task in a sample of young female adults overlapping with this study also provide indirect evidence of a particular importance of attentional deficits compared with inhibitory control deficits in this age group [36]. Attention-related networks were also altered in other alcohol-exposed samples studied with fMRI: Attention networks were among those altered in studies by Fan et al. [25] and Ware et al. [27]. In the latter study, FC alterations were associated with differences in attentional performance measures. The authors consequently conclude that the patterns observed (lower within-network, higher between-network FC) provide support for reduced attention network specialization and inefficiency [27]. Reduced FC between key regions of the salience network with other cognition-related networks were among the key findings by Little et al. [26]. There is further evidence of altered attention systems from magnetoencephalography [81] and diffusion tensor imaging [82].

### Group differences in networks related to cognitive control

To a lesser extent, we observed altered FC in parts of the fronto-parietal control network. This network is considered a flexible hub that interacts with other processing networks in order to orchestrate performance in a wide range of cognitive tasks [83]. Changes in fronto-parietal network FC were among those also observed in younger participants with FASD [25, 26]. There is further evidence of altered activity in these networks in children with FASD from task-based fMRI studies on inhibitory control [84] and working memory [85].

### Group differences in the default mode network

The default mode network (DMN), though classically reported as anti-correlated with task-positive cognitive networks [86], is considered to be involved in cognitive functions including task-switching and integration of information [87, 88]. There is evidence of regional differentiation within the DMN, with subdivisions subserving different cognitive functions [89]. Fan et al. observed altered FC in the anterior part of the DMN, discussed as subserving social perception, judgment, and self-referential processing in individuals with FASD; however, they found no changes within the posterior DMN [25]. Different from the preprint version of this analysis (i.e., not controlling for covariates in the primary models) [59], we did not find evidence of a strong predominance of group effects in either the anterior or posterior part of the DMN. Beyond that, there is further evidence of less regionally specific DMN dysfunction in FASD [32]. One single connection within the DMN was significant when correcting for multiple comparisons within the sub-network only, but not with global correction. Considering the distribution of effects within this network in comparison with the other networks, we consider this a potential outlier.

### Specific aspects of the statistical approach underlying main results

The main static FC analysis applied in this study follows a hierarchical statistical approach, partially based on HC statistics. This approach addresses the general limitations of functional neuroimaging analyses in relatively infrequent disorders such as FAS: Conventional high-dimensional FC analysis methods, such as frequently used mass-univariate statistical testing, carry the risk to report only a “tip of the iceberg” of true underlying alterations due to lower than optimal statistical power. There is an increased risk of both published findings being false-positive [90, 91] or false-negative findings [90]. Further in-depth discussions on this issue have been published [91–93]. Consequently, statistical thresholding of mass-univariate analyses (mainly for multiple-comparison correction) might in part explain ambiguous FC results in previous studies in children with FASD [25–27]. Such a conventional analysis approach as applied at the most detailed analysis level in this study did not yield significant group effects regarding single connections between regions of interest in this study when correcting for multiple comparisons across all connections. There are increasing efforts to report subthreshold effects in fMRI studies in order to facilitate better interpretation of underlying patterns [94–96]. An example is the additional presentation of unthresholded activation or connectivity maps [95, 97]. Our hierarchical approach with HC-based joint hypothesis tests [60, 61] at the network level might help avoid these shortcomings without sacrificing information from individual connections. Compared with conventional mass-univariate fMRI analyses, it avoids selectively reporting and interpreting few selected results that would pass a multiple-comparison threshold but might not well represent the true underlying effect in a medium-power setting. The finding of group effects in HC statistics not being observed in the conventional mass-univariate analysis might indicate that substantially larger samples are desirable in future rs-fMRI studies in neurodevelopmental disorders such as FAS. This is, however, limited by the rarity of these disorders [98]. Recent work in a related field further highlights the potential specific relevance of a network-perspective and limitations of group mean comparisons at the regional perspective: Segal et al. observed that structural brain alterations in individuals with mental disorders mainly converge at the network level while effects on the regional level are sparse and heterogeneous [99]. Please see the “Methods” section for a more detailed description of the HC-based multi-level approach and underlying rationale, as well as the “Potential limitations” section for further methodical aspects.

### No evidence of group differences in dynamic functional connectivity (additional exploratory analysis)

Our findings discussed so far are based on conventional static FC analyses. Clinical features typically observed in FASD include impulsivity or hyperactivity (including overlap/comorbidity with attention deficit hyperactivity disorder) [1, 3, 4]. These clinical features might suggest a potential dynamic or temporally changing nature of underlying neural disease mechanisms. This assumption is supported by dynamic FC alterations previously reported in ADHD [22–24]. Contrary to this assumption, we did not observe alterations of features representing non-stationarity in the exploratory time-resolved analysis of dynamical aspects of FC. In particular, compared to controls FAS participants did neither change more or less frequently between two putative FC states, nor did they remain in the different FC states for shorter or longer periods of times. Thus, we did not find evidence of altered dynamic interactions of brain regions of different networks. There is at least some evidence in children, that hyperactivity might be less severe in PAE compared with ADHD [100]. Though those findings cannot be directly translated to adult FAS participants, relatively lower hyperactivity might be the reason for the lack of dynamic FC alterations observed in our study. These findings are also generally in line with a previous rs-fMRI study reporting no alteration in regional temporal variance in less severely affected children with low levels of PAE [101].

### Potential limitations

Findings of this first FC analysis in adults with PAE are restricted to young adults (ages 18–32 years) with FAS. They do not necessarily translate to other age groups. Furthermore, findings do also not necessarily translate to other gradations across the FASD spectrum. Despite greater psychopathology, attention deficits, and impulsiveness compared with controls, a recent study did not find network-based FC alterations in a population of adolescents with a wide range of PAE, i.e., less severely exposed individuals [102]. Adult participants with FAS in this sample had been diagnosed during their childhood using the Majewski criteria [37] then widely used in German-speaking countries. These criteria did not gain widespread use in other countries [38]. Although they generally reflect the full clinical picture of FAS (see also Additional_file_1.pdf: supplementary methods), disease severity cannot be exactly mapped onto newer diagnostic criteria [2]. Explicit information about the presence and quantity of maternal alcohol exposure is not available in this sample. This represents a more general limitation of FAS diagnosis both, in research and clinical care: As reflected by current clinical guidelines [8, 103–105], FAS can be diagnosed based on the typical clinical picture and does not require explicit knowledge about prenatal alcohol exposure. Similarly, there is a possibility of unknown sub-clinical prenatal alcohol exposure in the control group, potentially reducing the sensitivity for FC group differences. While occasional alcohol consumption has been reported in 14%, regular alcohol consumption during pregnancy has been relatively rarely reported (around 1%) in Germany [106]. Social and psychopathological characterization of the participants and their families is limited: no information about socioeconomic status is available. Socioeconomic inequalities such as parental income, educational status, or neighborhood context have been related to differences in structural brain development and to disrupted development of cognitive abilities [107]. Early SES disadvantage in childhood has been associated with altered resting-state functional connectivity of brain networks involved in cognition [108]. Children with FASD are frequently exposed to such adverse experiences when growing up [109–111]. In addition to the influence of prenatal alcohol exposure itself, SES might therefore pose an independent factor that could have interfered with brain development in our sample [110]. Future research is needed to highlight the effects of the additional burden imposed upon FASD subjects by socioeconomic disadvantage and the associated implications on brain structure and activity in affected individuals. Information on psychiatric comorbidity for the exclusion criteria is based on anamnestic information and a screening tool (SSQ), but not on detailed assessment within this study or on previously assigned ICD or DSM diagnostic codes. We did not include total brain volume and IQ in the statistical models of FC alterations, since both reduced IQs [1] and reduced total brain volumes [112, 113] are considered disease features of FAS with the potential for overcorrection if included as covariates [114].

Taking differences in head motion into account in rs-fMRI studies in clinical populations is a matter of ongoing critical debate [115]. Although we have taken precautions to minimize head motion, excluded participants with excessive head motion, include head motion in the analyses and even though measures did not differ significantly between groups, it cannot be excluded that parts of the results are movement-related [53, 115]. The state-of-the-art motion correction methods during data pre-processing are very similar to those showing a particularly good performance in an additional large-scale benchmarking study of rs-fMRI motion correction strategies [115]. We have also included head motion as a covariate in the main FC group analyses at the level of individual connections to further take a potential movement bias into account. This also means that connections with different distances between brain regions were modelled separately at this stage. We refrained from including global signal regression since it may confound the directionality of FC estimates and aggravate potential distance-related effects of residual head motion [115]. In this, context, it should however also be considered that motor restlessness itself is a disease feature in FAS [116].

Statistical power of the final step of the hierarchical analysis approach (resolving FC alterations to single connections between pairs of regions) is potentially limited by the high dimensionality of the underlying atlas. This notion is also supported by the distribution of effect size estimates for group differences of individual connections. This atlas resolution was chosen because it is extensively validated [56] and aims to optimally reflect the brain’s functional architecture [56, 117]. The HC-based approach was chosen here instead of averaging FC estimates to allow global and network-wise inference while maintaining the advantages of the high-resolution atlas. Findings are limited to the cortex and do not include subcortical gray matter nuclei within these networks [118, 119]. It has to be noted that there is an ongoing debate about functional network nomenclature, so that the networks described here [56] may deviate from studies using other atlases [120].

Though widely used in other research areas with high-dimensional data [60, 63], the HC statistic has only recently been introduced to fMRI [64, 65, 96]. The HC statistic primarily assumes statistically independent features, since correlations among features can lead to unbalanced *p*-value histograms, however without expecting peaks in the first histogram bin (low *p*-values). It has thus been argued that the influence of correlations among features is negligible when the underlying histograms show typical behavior [62]. In addition to the HC statistic, we therefore visually interpreted the underlying *p*-value histograms as a plausibility control and observed well-behaved *p*-value histograms in the global and within-network analysis and to a slightly lesser degree in the between-network analysis. The HC-based global hypothesis test does not directly result in *p*-values but in a primary decision to reject/retain the corresponding global null hypothesis. However, *p*-values for typical HC values have been approximated [121]. Even when using these approximated *p*-values, thus departing from the notion of the original HC statistic, main results would be statistically significant at the *p* < 0.05 level or stricter *p*-values.

Dynamic or time-resolved FC analysis is a promising, already widely used, yet still evolving rs-fMRI analysis method [15–17]. Thus, there is currently a relatively high methodological variability [15, 17]. Here, we adopted a widely used sliding window approach [17] and refrained from extensively exploring analysis settings in order to avoid false-positive findings [122]. Hence, there is a risk to miss group differences of FC dynamics which might have been uncovered with other, less well-established dynamic FC analysis approaches [15, 17, 123]. Furthermore, FC states themselves, which were estimated in the entire sample here, might be intrinsically different in both groups. This might be addressed by separate FC state estimation, however will need in the future more advanced methodology for subsequent actual group comparison. Similar to methodological heterogeneity and open methodological questions, there is still no consensus of connectivity states to be expected in a normal population. However, a relatively small number of FC states partially reflecting changing interactions of parts of the DMN, similar to those observed here (Additional_file_1.pdf: Supplementary Fig. 2) has been repeatedly reported [17, 124]. Silhouette values (Additional_file_1.pdf: Supplementary Fig. 1) indicate that clusters observed in our analysis may not be well separated. Thus, these two clusters capture dynamic FC changes as a model but might not represent truly discrete FC states in a neurobiological sense. Cluster frequencies suggest a high inter-subject variability. Despite these general limitations of this evolving methodology, we believe that our exploratory approach can be a starting point for further investigations on dynamic FC in FAS and other neurodevelopmental disorders.

## Conclusions

We observed altered FC in cognition-related brain networks in young adults with FAS. Using a HC-based statistical approach, this study provides evidence of the existence of at least rare and weak effects (i.e., FC differences between participants with FAS and controls) widely distributed across a majority of these networks, potentially underlying the diversity of cognitive deficits in these individuals. Findings were pronounced in attention-related sub-networks, which is in line with substantial attentional deficits previously reported. Relevant for comparisons with previous studies is that—in contrast with network-level results—the most detailed analysis level using a more conventional mass-univariate approach did not identify significant group differences. Thus, findings could not be resolved to single functional connections. An exploratory time-resolved analysis did also not identify altered FC dynamics and could thus not explain reduced impulse control and attention deficits which have been frequently reported in FAS.

### Supplementary Information

**Additional file 1.**Document containing all supplementary methods information (rationale of the Majewski criteria for FAS, MRI data pre-processing), supplementary figures and tables (Supplementary Table 1: Group comparison of head motion; Supplementary Figure 1: Goodness-of-fit statistics for different k-means clustering solutions; Supplementary Figure 2: Connectivity matrices representing the two putative functional connectivity (FC) states in the optimal clustering solution of the time-resolved analysis)

**Additional file 2.**Detailed naming of regions of interest and order key (overall and within networks)

**Additional file 3.**Static functional connectivity differences (differences of z-transformed correlation coefficients averages across groups) between the full set of regions of interest as visualized in Figure 1C.

**Additional file 4.**Standardized effect sizes (partial eta^2^) of static functional connectivity differences as visualized in Figure 1D and ordered identical to additional file 3.

## Data Availability

On request to the authors, further intermediate data on a level independent from the individual participants can be shared. Due to German data protection regulations and to safeguard subject confidentiality, data on the level of individual participants cannot be made available (no participant consent for sharing these primary data). Software and statistical algorithms used for these analyses are freely available as referenced in the “Methods” section. Further in-house code was used for individual aspects of data handling only and could be made available to other researchers on reasonable request.

## Abbreviations

ADHD: Attention deficit hyperactivity disorder
BIDS: Brain Imaging Data Structure
DMN: Default mode network
EHI: Edinburgh Handedness Inventory
FAS: Fetal alcohol syndrome
FASD: Fetal alcohol spectrum disorders
FC: Functional connectivity
FD: Frame-wise displacement
FDR: False discovery rate
fMRI: Functional magnetic resonance imaging
FRC: Frequency of observing each cluster
FWHM: Full width at half maximum
HC: Higher criticism
MDT: Mean dwell time
MRI: Magnetic resonance imaging
NT: Number of transitions
PAE: Prenatal alcohol exposure
rs-fMRI: Resting-state functional magnetic resonance imaging
SSQ: Stamm-Screening questionnaire
TFE: Turbo field echo
TMT: Trail-making task
